# Repurposing salmon calcitonin for glioblastoma treatment: Targeting Yes-associated Protein (YAP)/ Transcriptional co‑activator with PDZ‑binding motif (TAZ) via hippo pathway activation

**DOI:** 10.1093/noajnl/vdag152

**Published:** 2026-06-05

**Authors:** Jayita Goswami, Prithviraj Uttarasili, Lakshay Garg, Prasasvi Kaval Reddy, Abhishek Chowdhury, Subhrodeep Saha, Jayanta Chatterjee, Anand Srivastava, Kumaravel Somasundaram

**Affiliations:** Department of Microbiology and Cell Biology, Indian Institute of Science, Bangalore, India; Molecular Biophysics Unit, Indian Institute of Science, Bangalore, India; Department of Microbiology and Cell Biology, Indian Institute of Science, Bangalore, India; Department of Microbiology and Cell Biology, Indian Institute of Science, Bangalore, India; Department of Microbiology and Cell Biology, Indian Institute of Science, Bangalore, India; Molecular Biophysics Unit, Indian Institute of Science, Bangalore, India; Molecular Biophysics Unit, Indian Institute of Science, Bangalore, India; Molecular Biophysics Unit, Indian Institute of Science, Bangalore, India; Department of Microbiology and Cell Biology, Indian Institute of Science, Bangalore, India

**Keywords:** calcitonin receptor, G protein-coupled receptor, glioblastoma, hippo signaling, salmon calcitonin

## Abstract

**Background:**

Glioblastoma (GBM) remains a deadly brain tumor. Activation of the wild-type calcitonin receptor (CTR) by salmon calcitonin (sCT) suppresses glioma growth, whereas patient-derived CTR mutants drive tumor progression and correlate with poor survival. This study delineates the mechanisms underlying sCT-mediated tumor suppression and reveals structural defects in oncogenic CTR variants.

**Methods:**

Proteome analysis of sCT-treated glioma cells was done to dissect the altered signaling. Multiple experimental approaches were used to elucidate the signaling behind CTR-dependent Hippo pathway activation. The therapeutic efficacy of sCT on the growth of human/murine glioma stem-like cells (GSC)-initiated tumors in an orthotopic mouse glioma model was assessed. Extensive microsecond-scale all-atom molecular dynamics simulations were performed to elucidate the structural impairments in CTR mutants.

**Results:**

sCT treatment activated Hippo signaling as demonstrated by enhanced phosphorylation and subsequent degradation of YAP/TAZ and suppressed glioma growth through cAMP/PKA/LATS1 signaling in vitro. However, GSCs expressing a phosphorylation-resistant YAP mutant were refractory to sCT treatment *in vitro*. Both human and murine GSCs exhibited elevated CTR expression, and intranasal administration of sCT effectively inhibited the growth of GSC-initiated tumors. Furthermore, molecular dynamics simulations revealed structural perturbations in CTR interactions with either CT or the Gα subunit.

**Conclusions:**

We establish that activation of the sCT-CTR axis suppresses glioma growth by inhibiting YAP/TAZ through Hippo pathway activation. We further delineate the structural aberrations responsible for the functional loss in patient-derived CTR mutants. Thus, we present compelling evidence supporting the therapeutic repurposing of calcitonin for GBM treatment.

## Graphical Abstract

**Graphical Abstract:**
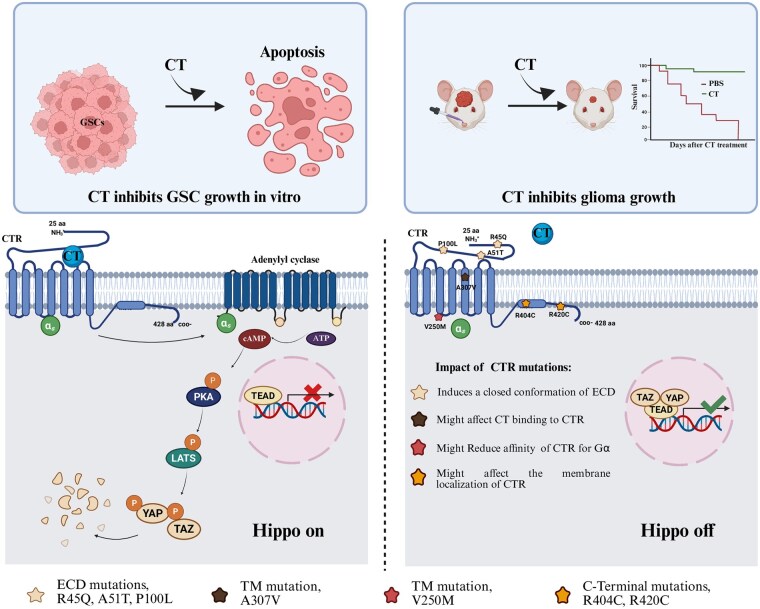

Key Points

- sCT/CTR axis targets YAP/TAZ through cAMP-PKA-LATS1-dependent Hippo pathway activation.
- Glioma stem-like cells express higher levels of CTR, and intranasal delivery of sCT suppresses patient-derived GSC-initiated tumors in a mouse model.
- AAMD simulations uncover structural perturbations in the CTR mutants.

Importance of the Study

Glioblastoma remains incurable and lethal. Cancer stem cells (CSCs), which underlie tumor initiation, therapeutic resistance, and recurrence, are central contributors to their adverse clinical outcomes. Our previous work demonstrated that GBMs harboring loss-of-function mutations or reduced expression of the calcitonin receptor (CTR) exhibit greater aggressiveness, whereas activation of the calcitonin (CT)/wild-type CTR axis suppresses glioma growth. In the present study, we demonstrate that sCT activates the Hippo signaling pathway, leading to the suppression of oncogenic YAP/TAZ activity. Glioma stem-like cells (GSCs), the CSC population in gliomas, display markedly elevated CTR expression. Notably, intranasal administration of sCT significantly inhibited tumor growth driven by patient-derived and murine GSCs in orthotopic mouse models. Furthermore, microsecond-scale all-atom molecular dynamics simulations revealed structural perturbations in loss-of-function CTR mutants. Together, these findings provide compelling evidence in support of repurposing sCT as a targeted, noninvasive therapeutic strategy for GBM.

G protein-coupled receptors (GPCRs), which represent the largest protein family, have been implicated in several diseases, such as cancer, and others.^1^ GPCRs have been considered targets for developing drugs as they control a wide range of physiological processes. While 34% of FDA-approved drugs target GPCRs, only 8 of them have been developed as anticancer drugs.^2^ Aberrant gene expression due to copy number aberrations, epigenetic mechanisms, and genetic mutations in GPCRs has been shown to induce ligand-dependent or independent constitutive signaling, contributing to oncogenesis.^2-6^

Calcitonin receptor (CTR), a member of the class B family of GPCRs, binds calcitonin (CT), a neuroactive peptide, to maintain calcium homeostasis in bone and kidney.^7^ While the CTR expression has been demonstrated in many cancers, the role of CTR in cancer initiation and development remains to be established. While CT treatment has been shown to inhibit apoptosis and promote prostate tumor growth,^8^ the CT/CTR axis inhibited the growth of breast cancer, glioma, and giant cell tumors.^9-11^ Loss-of-function mutations in glioblastoma (GBM) identified a subgroup with a poor prognosis.^11^ The glioma cell growth inhibition by sCT treatment in a WT CTR-dependent manner is correlated with inhibition of JNK, ERK, and AKT phosphorylation.^11^ WT CTR, but not CTR mutants, inhibited the Ras-mediated transformation of immortalized astrocytes.^11^ However, the precise mechanism of sCT-mediated growth inhibition and the signaling downstream of CTR remain to be elucidated.

An integrative analysis of TCGA cancer datasets revealed that approximately 20% of sequenced human tumors harbor mutations in genes encoding GPCRs.^2^^,^^12^ A survival correlation analysis of mutated genes and pathways identified that patients with a defective “neuroactive ligand interaction pathway” had a worse prognosis.^11^ A focused investigation revealed that CTR with patient-derived mutations failed to respond to sCT and inhibit glioma growth.^11^ Recently, a near-atomic-resolution structure of CT-bound CTR was obtained.^13^ CT binding to extracellular loops alters the structure of the transmembrane domain, facilitating the stimulatory interaction between Gα and the intracellular loops of CTR.^13-17^ However, the impact of loss-of-function mutations on the CTR structure is unclear.

In this study, we found that the sCT/CTR axis inhibits oncogenic transcriptional coactivators, YAP/TAZ, by activating the Hippo pathway via cAMP/PKA/LATS1 signaling. Intranasal delivery of sCT inhibited the growth of human and murine glioma-like stem cells (GSCs)-initiated tumors in an intracranial mouse model. Large-scale all-atom molecular dynamics (AAMD) simulation studies revealed that each patient-derived mutant significantly alters CTR packing and its conformational dynamics. Depending on the location of the mutations in the CTR, the mutant system either compromises CT binding, Gα binding, or the strength of membrane lipid interactions.

## Methods

### Experimental Model and Subject Details

The animal studies were conducted on 6 to 8-week-old female C57BL/6J mice and athymic nude mice (NIH nu/nu), with approval from the Institute’s Ethical Committee for Animal Experimentation under Project Number CAF/Ethics/743/2020. The animals were maintained under specific pathogen-free conditions with a 12-hour light-dark cycle, controlled temperature and humidity, and provided unlimited access to a standard diet. All the experiments were conducted during the light phase of the cycle. Sample sizes were determined based on prior studies. Animals were stratified by IVIS imaging to ensure comparable average tumor burden prior to treatment, randomly assigned to experimental groups, and monitored daily. Humane endpoints included >20% body weight loss, impaired mobility, seizure activity, or signs of distress, upon which animals were euthanized in accordance with institutional animal ethics guidelines.

### Plasmids

8xGTIIC-luciferase (TEAD promoter Luc plasmid), mEGFP-N1, mEGFP-N1-YAP, and mEGFP-N1-YAPS127A, CRE-Luc plasmids were bought from Addgene, USA (#43806). The CTGF Promoter Luc construct was created in our lab.^18^

### RPPA for the Identification of Differential Protein Expression upon sCT Treatment

sCT-treated (300 nM) and untreated LN229 cells were lysed using RPPA lysis buffer. The resulting lysates were serially diluted in five twofold steps using the same lysis buffer and then printed onto nitrocellulose-coated slides using an Aushon Biosystem 2470 arrayer. Afterwards, the slides were probed with 441 validated primary antibodies, followed by detection using suitable biotinylated secondary antibodies. The slides were scanned, analyzed, and quantified using Array-Pro Analyzer software (Media Cybernetics) to obtain spot intensity data (level 1 data). Signal visualization was achieved through a secondary streptavidin-conjugated HRP antibody and DAB colorimetric reaction. The list of the 441 antibodies utilized is provided at https://www.mdanderson.org/research/research-resources/core-facilities/functional-proteomics-rppa-core/education-and-references.html. The full RPPA profiling data is publicly available at the GEO database, GSE319121.

### Immunofluorescence Staining of Fixed Cells

Cells were seeded on coverslips in 12-well plates and allowed to adhere. Upon completion of the experiment, the cells were fixed using 4% paraformaldehyde for 15 minutes at room temperature and permeabilized with PBS containing 0.25% Triton-X100. Subsequently, the cells were washed with PBS and blocked in a PBS solution containing 1% BSA, 0.3% Triton X-100, and 5% goat serum for 2 hours at room temperature. After blocking, primary antibodies diluted in the blocking buffer were added to the coverslips and incubated at 4°C overnight. Following primary antibody incubation, cells were washed three times with PBS for 5 minutes each. Fluorescence-conjugated secondary antibodies, prepared in blocking buffer, were added to the cells for 3 hours, followed by another round of washing with PBS. The cells were counterstained with DAPI (1 µg/mL) for 5 minutes at room temperature. Finally, coverslips were mounted onto glass slides using an anti-fade reagent as a mounting medium and imaged using a Zeiss LSM 880 confocal microscope.

### Limiting Dilution Assay

Sphere formation assays were conducted by plating 1, 10, 50, 100, and 200 individual GSCs per well in 10 wells each of a 96-well plate. Single-cell plating was confirmed through microscope. Cells were cultured in serum-free neurobasal medium supplemented with growth factors like l-glutamine, heparin, B27 supplement, N2 supplement, rhEGF, rhFGF-basic, and antibiotics and maintained at 37°C in a humidified incubator with 5% CO_2_. PBS or sCT (100 nM) was added to the wells 12 hours after plating and replenished every 24 hours by partial medium replacement with fresh growth factor-containing medium. Over the subsequent 5-7 days, the presence or absence of sphere formation was monitored. The count of wells where sphere formation did not occur was recorded and plotted against the initial number of cells per well. Utilizing the Extreme Limiting Dilution Assay (ELDA) software, data analysis was performed (https://bioinf.wehi.edu.au/software/elda/).

### Other Methods and Materials

The details of other methods and materials used, as described below, are given in the Supplementary Section: Cell lines, Neurosphere culturing, GSC Differentiation/dedifferentiation, Lentivirus preparation and transduction of cells, Luciferase and beta-galactosidase (β-gal) assay, RNA isolation, cDNA conversion, and Real-qPCR and the list of primers, Western Blotting, Apoptosis assay, Colony Formation assay, Cell Viability assay, Intracranial injection, *In vivo* Imaging, Hematoxylin and Eosin staining, Survival Analysis, Quantification and Statistical Analysis, Molecular Modeling of CTR, Insilico reconstitution of full-length CTR from structurally solved fragments, *In silico* reconstitution of mutant CTR with binding partners, and molecular dynamics simulation details.

## Results

### sCT/CTR Signaling Activates the Hippo Pathway to Inhibit YAP/TAZ and Suppresses Glioma Growth

**Figure 1.**
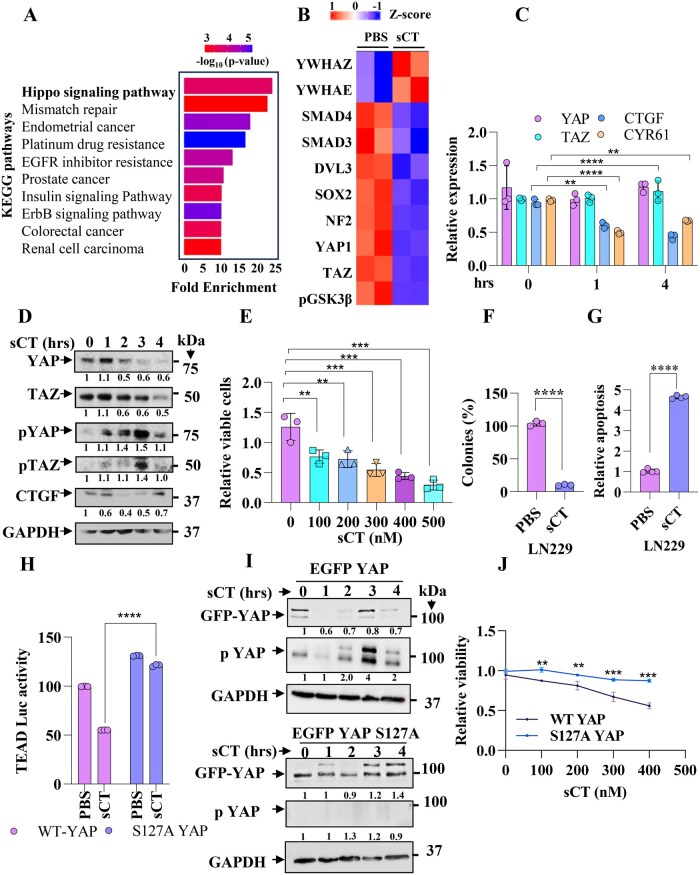
sCT inhibits YAP and TAZ protein. (A) Differentially expressed proteins identified through Reverse Phase Protein Array (RPPA) analysis in sCT-treated LN229 cells and subsequently analyzed using DAVID for KEGG pathway enrichment reveal significant enrichment (*P* < .05) of the Hippo pathway following sCT treatment (300 nM). (B) Heatmap showing differentially regulated (|log_2_FC| ≥ 0.58, *P* < .05) genes of the Hippo pathway after sCT treatment, assessed from RPPA data. Red and blue represent upregulated and downregulated genes under the sCT-treated condition. (C) RT-qPCR analysis shows YAP, TAZ, CTGF, and CYR61 transcript levels upon sCT treatment (300 nM) in the LN229 cell line for the indicated time points (two-way ANOVA *n* = 3/group). (D) Western blotting shows YAP, TAZ, pYAP, pTAZ, and CTGF upon sCT treatment for the indicated time points. (E) MTT assay showing the viability of LN229 cells at the indicated concentrations of sCT (nM) after 72 hours of treatment (one-way ANOVA *n* = 4/group). (F) Quantification of colony formation under PBS and sCT-treated (100 nM) conditions (t test; *n* = 4/group). (G) Quantification of apoptotic cells by annexin V and PI staining after sCT (300 nM) treatment for 72 hours (t test; *n* = 4/group). (H) The plot shows TEAD Luc activity from LN229 cells co-expressing WT YAP or constitutively active YAP S127A (two-way ANOVA; *n* = 3/group) treated with PBS or sCT (300 nM) for 4 hours. (I) Western blot analysis showing the effect of sCT (300 nM) on LN229 cells expressing WT or S127A mutant YAP at the indicated time points following treatment. (J) The graph shows the effect of indicated doses of sCT (nM) on the viability of LN229 cells expressing WT or S127A YAP (two-way ANOVA; *n* = 3/group). Data are presented as mean ± SD; *P < .05, **P < .01, ***P < .001, ****P < .0001.

We previously demonstrated that the CT/CTR signaling axis exerts a growth-suppressive effect in glioma.^11^ To elucidate the downstream molecular events and effector proteins mediating this response, we subjected extracts of sCT-treated LN229 glioma cells to Reverse Phase Protein Array (RPPA) profiling. This unbiased analysis identified a spectrum of regulated proteins that were subjected to Gene Ontology (GO). GO analysis revealed a significant enrichment of the Hippo signaling pathway (Figure 1A; Supplementary Table S1), suggesting that the CT/CTR axis modulates Hippo pathway activity. The Hippo pathway is an evolutionarily conserved signaling cascade that governs organ size and tissue homeostasis, and its deregulation is implicated in many diseases, including cancer.^19^ Mechanistically, pathway activation involves MST1/2-LATS1/2 kinase-cascade-dependent phosphorylation of YAP/TAZ, which is subsequently retained in the cytoplasm by 14-3-3 proteins, followed by ubiquitin-mediated proteasomal degradation.^20^

RPPA data analysis demonstrated a marked reduction in YAP, TAZ, and SOX2 (an established YAP target), concomitant with increased expression of YWHAZ and YWHAE, 2 members of the 14-3-3 protein family in sCT-treated cells, which supported the presence of an activated Hippo pathway (Figure 1B; Supplementary Table S2). Hippo pathway activation was confirmed by significant decrease in TEAD-Luc and CTGF-Luc promoter (YAP/TAZ-dependent reporters) activity (Supplementary Figure S1A-D), downregulation of the canonical YAP/TAZ target genes, CTGF and CYR61 with no change in YAP and TAZ transcript levels (Figure 1C; Supplementary Figure S1E), decreased abundance of total YAP and TAZ with a simultaneous enhanced phosphorylation of both proteins (Figure 1D; Supplementary Figure S1F), diminished nuclear localization of YAP (Supplementary Figure S1G and H), and reduced CTGF protein levels (Figure 1D; Supplementary Figure S1F) in sCT-treated LN229 and T98G glioma cell lines.

Next, we found that sCT treatment significantly suppressed proliferation, inhibited colony-forming ability, and induced apoptosis (Figure 1E-G; Supplementary Figure S1I) but not in CTR-silenced cells (Supplementary Figure 2A-C), establishing the requirement of CTR for the growth-inhibitory activity of sCT. Further, CTR knockdown abrogated sCT-induced Hippo pathway activation, as evidenced by the inefficient suppression of YAP/TAZ-dependent reporter activity (Supplementary Figure S2D) and the absence of reductions in total YAP/TAZ or increases in pYAP/pTAZ protein levels (Supplementary Figure S2E). Additionally, to confirm the requirement of YAP/TAZ targeting, we used a phosphorylation-resistant YAP mutant (S127A), which constitutively drives transcriptional activation and is refractory to Hippo pathway-mediated inhibition.^21^ In cells expressing the S127A YAP mutant, sCT treatment failed to reduce TEAD-Luc reporter activity, diminish total YAP, enhance pYAP, or impair proliferation (Figure 1H-J).

The CT/CTR axis is also known to regulate intracellular calcium signaling.^22^ Further, intracellular calcium levels also known to regulate YAP activity.^23^ Hence, we examined whether calcium signaling contributes to sCT-mediated inhibition of TEAD transcriptional activity using the intracellular calcium chelator BAPTA-AM. Treatment with BAPTA-AM significantly reduced basal TEAD-luciferase activity, suggesting a role for intracellular calcium in maintaining TEAD activity, in good correlation with earlier reports (Supplementary Figure S2F, compare bar 2 with 1). However, sCT treatment further suppressed TEAD-luciferase activity even in the presence of BAPTA-AM (Supplementary Figure S2F, compare bar 4 with 3), indicating that sCT-mediated inhibition of TEAD activity is largely independent of intracellular calcium signaling. Collectively, these findings establish that the sCT/CTR axis executes a tumor-suppressive effect by the Hippo signaling activation and the subsequent YAP/TAZ targeting.

### Hippo Pathway Activation by CT/CTR Involves cAMP/PKA/LATS1 Signaling

**Figure 2.**
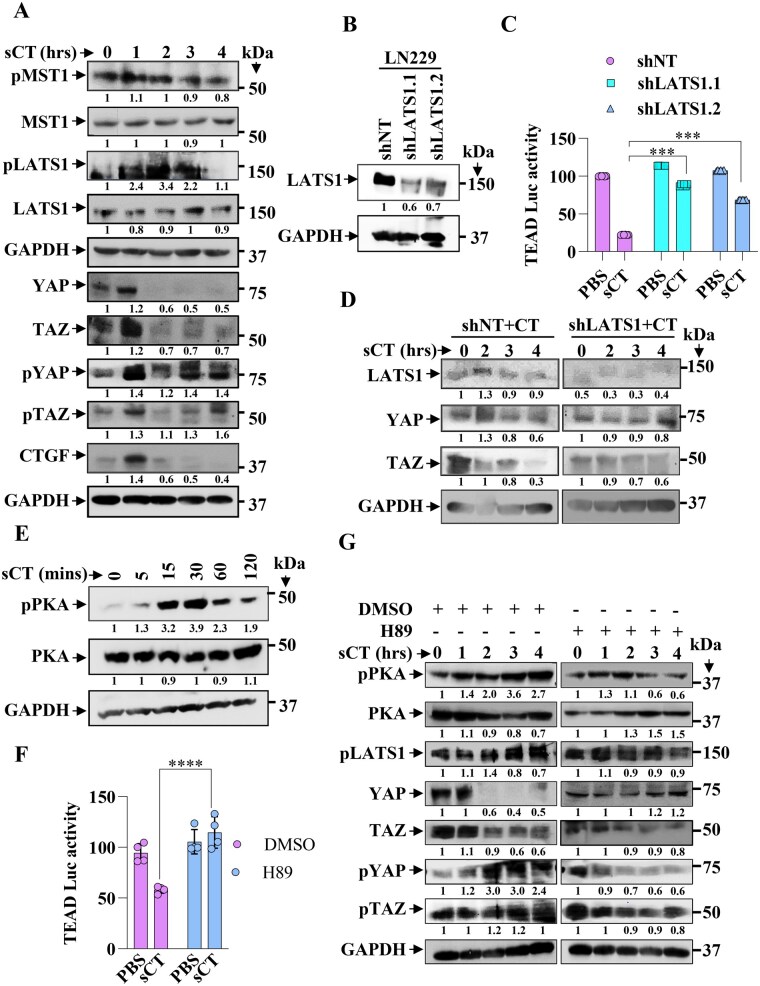
sCT modulates YAP and TAZ in a cAMP-PKA-LATS1-dependent manner. (A) Western blot shows the effect of sCT treatment (300 nM) on YAP, TAZ, and the upstream regulatory kinases MST1 and LATS1 in LN229 cells. (B) Western blot shows LATS1 knockdown using two shRNAs in LN229 cells. (C) LN229/shNT and LN229/shLATS1 cells were transfected with the TEAD luc construct, and the luciferase activity was determined after 4 hours of sCT treatment (300 nM). (D) Western blot shows the levels of YAP and TAZ upon CT treatment (300 nM) under shNT and shLATS1 conditions at the indicated time-points. (E) Western blotting shows the activation status of PKA in a time-dependent manner upon sCT treatment (300 nM). (F) LN229 cells expressing the TEAD luciferase reporter were pretreated with vehicle (DMSO) or the PKA inhibitor H89 (10 µM) for 2 hours prior to sCT (300 nM) treatment for 4 hours. The cell extracts were used to determine the relative TEAD Luc activity (two-way ANOVA; *n* = 4/group). (G) The western blot shows the effect of sCT (300 nM) on the PKA, LATS1, YAP, and TAZ in DMSO or H89-pretreated (10 µM) LN229 cells at the indicated time-points. Data are presented as mean ± SD; *P < .05, **P < .01, ***P < .001, ****P < .0001.

To elucidate the molecular basis of Hippo pathway activation by sCT, we examined the phosphorylation status of the upstream kinases MST1 and LATS1. sCT treatment significantly increased phosphorylated LATS1 (pLATS1) levels but not total LATS1 protein levels (Figure 2A), with no change in phosphorylated or total MST1, indicating that sCT activates the Hippo pathway at the LATS1 level and is MST1-independent. In glioma cells with LATS1 knockdown, sCT treatment failed to suppress TEAD-luciferase reporter activity, decrease total YAP protein, reduce nuclear YAP localization, or inhibit proliferation (Figure 2B-D; Supplementary Figure S2G and H). Complementary pharmacological inhibition of LATS1/2 with TRULI similarly abolished the effects of sCT, including repression of TEAD reporter activity, downregulation of YAP/TAZ, increase in pYAP and pTAZ levels, exclusion of YAP from the nucleus, and inhibition of cell growth (Supplementary Figure S3A-D). These findings establish LATS1 as a key mediator of CT/CTR-induced Hippo pathway activation, underscoring its essential role in transducing the anti-tumor effects of sCT signaling in glioma cells.

The cAMP-dependent protein kinase A (PKA) axis has been reported to facilitate Hippo pathway activation by phosphorylating LATS1.^24^^,^^25^ Aptly, sCT treatment of glioma cells resulted in a marked increase in phosphorylated PKA (pPKA) levels, without affecting the total abundance of PKA protein (Figure 2E). Importantly, pharmacological inhibition of PKA with H89 abrogated sCT-mediated Hippo activation and suppression of cell proliferation (Figure 2F and G; Supplementary Figure S3E and F). As CT/CTR signaling is known to stimulate adenylate cyclase,^26^ we next examined intracellular cAMP levels and observed a significant elevation following sCT treatment (Supplementary Figure S3G). Furthermore, the addition of forskolin (FSK), a direct activator of adenylate cyclase, recapitulated the effects of sCT treatment on glioma cells (Supplementary Figure S4A-E). Collectively, these findings define a mechanistic framework in which CT/CTR signaling activates the Hippo pathway through a cAMP/PKA/LATS1 cascade, resulting in the suppression of YAP/TAZ transcriptional activity and inhibition of glioma cell proliferation.

### *GSCs Express Elevated Levels of CTR, and sCT Treatment Inhibits Their Growth* in Vitro *and GSC-Initiated Tumors* in Vivo

**Figure 3.**
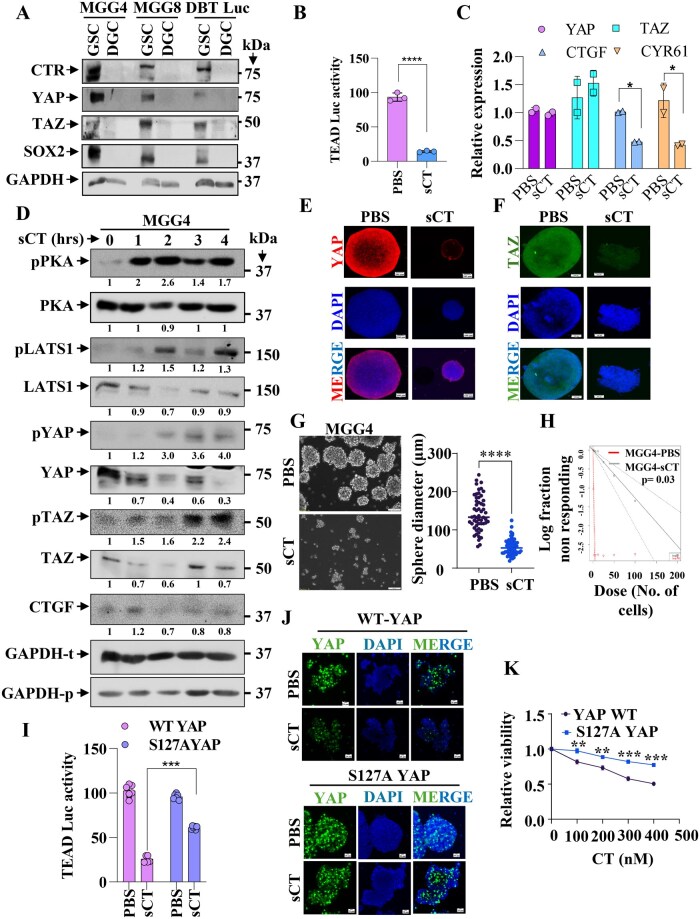
GSCs are better suited for sCT-mediated growth inhibition. (A) Western blotting shows differential expression of YAP, TAZ, and CTR in GSC vs. DGC of MGG4, MGG8, and DBT-Luc. (B) TEAD-Luc activity in MGG4 cells following PBS or sCT treatment (300 nM) for 4 hours (t test: *n* = 3/group). (C) RT-qPCR analysis shows YAP, TAZ, CTGF, and CYR61 transcript levels upon sCT treatment (300 nM) in the MGG4 cells (t test; *n* = 2/group). (D) Western blot showing the effect of sCT treatment (300 nM) on YAP, TAZ, and the upstream regulatory kinases MST1 and LATS1 in MGG4 cells at the indicated time points. While GAPDH (above) serves as a control for total-protein western blots, GAPDH (below) serves as a control for phospho-protein western blots. (E, F) Confocal microscopy analysis shows YAP and TAZ levels in MGG4 spheres with and without sCT treatment (300 nM) for 4 hours. Magnification x20, scale bar 100 µm. The merged images are shown for representation. (G, H) The sphere formation assay and the Limiting Dilution Assay show the effect of sCT (300 nM) on the growth of MGG4 cells. (I) The plot shows TEAD Luc activity from LN229 cells expressing WT YAP or constitutively active YAP S127A upon treatment with PBS or sCT (two-way ANOVA; *n* = 6/group) for 4 hours. (J) Confocal imaging shows the effect of sCT (300 nM) on MGG4 spheres expressing WT-YAP or S127A YAP for 4 hours. (K) The graph shows the effect of sCT (nM) on the viability of LN229 cells expressing WT or S127A YAP (two-way ANOVA; *n* = 3/group) after 72 hours. Data are presented as mean ± SD; *P < .05, **P < .01, ***P < .001, ****P < .0001.

GSCs, though representing a minor subpopulation GBM, are crucial for tumor initiation, maintenance, therapeutic resistance, and recurrence.^27^ Patient-derived human GSCs and murine GSCs showed elevated expression of the CTR and YAP/TAZ compared to matched differentiated glioma cells (DGCs) (Figure 3A). Consistent with their stem-like identity, GSCs from MGG8 and other models expressed higher levels of glioma-reprogramming factors (SOX2, SALL2, and POU3F2) than their matched DGCs (Supplementary Figure S5A). The high expression of CTR and YAP/TAZ underscores the vulnerability of GSCs to therapeutic strategies that activate the Hippo pathway. Indeed, sCT treatment of human and murine GSCs activated the Hippo pathway in GSC lines, MGG4 (Figure 3B-D), GB3 (Supplementary Figure S5B-D), MGG8 (Supplementary Figure S6A), and DBT-Luc (Supplementary Figure S6E). Additionally, sCT treatment inhibited the growth of human GSCs, MGG4 (Figure 3E-H), GB3 (Supplementary Figure S5E-G), MGG8 (Supplementary Figure S6B-D), and murine GSC, DBT-Luc (Supplementary Figure S6F-H) as seen from the neurosphere growth assay and limiting dilution assay. We also demonstrate that GSCs are more susceptible to growth inhibition following sCT treatment compared to a matched DGC (Supplementary Figure S5H). Further, MGG4 GSCs expressing the S127A-YAP mutant are found to be resistant to sCT-mediated GSC growth inhibition due to inefficient TEAD-Luc inhibition (Figure 3I), and persistent YAP abundance observed by confocal microscopy (Figure 3J) and continued proliferation (Figure 3K) in sCT-treated cells. These results indicate that GSCs can be targeted specifically by sCT-dependent activation of the Hippo pathway and inhibition of YAP/TAZ.

**Figure 4.**
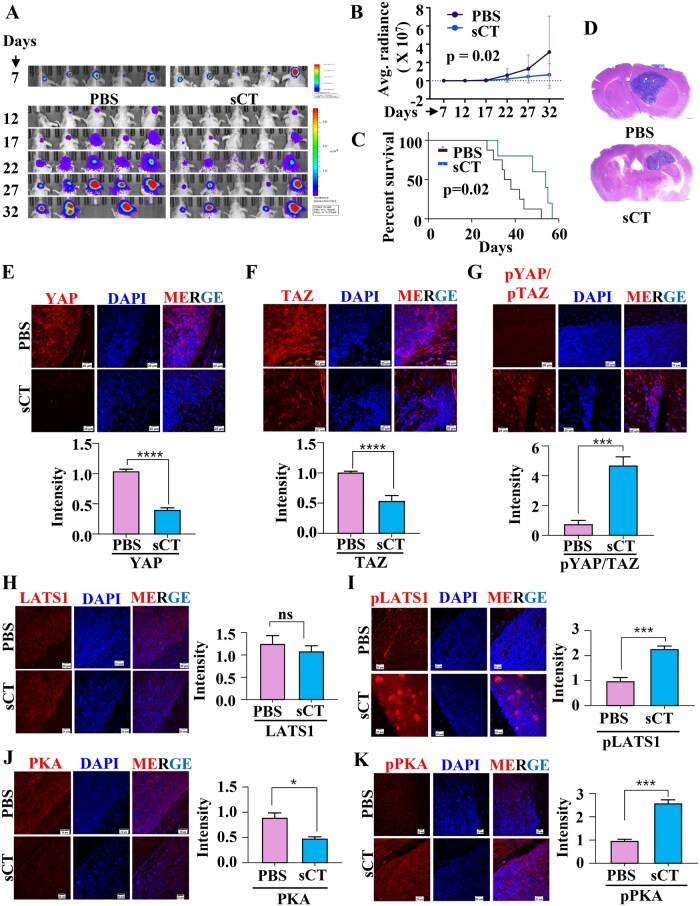
Intranasal sCT administration inhibits MGG4-Luc GSC-derived tumors in nude mice. (A) 0.15 × 10^6^ MGG4-Luc GSCs were injected intracranially into the brain of nude mice. The tumor was allowed to grow for seven days, after which sCT was administered intranasally at a dose of 2 IU/animal twice daily. Representative images of the animals’ in vivo bioluminescence were taken every 5 days from the date of injection. (B) The average radiance efficiency between the PBS and sCT-administered groups is plotted (two-way ANOVA; *n* = 8 in PBS group and *n* = 7 in sCT-treated group). (C) The Kaplan-Meier graph shows the survival difference between the two groups of mice that were administered PBS or sCT. (D) Hematoxylin and Eosin staining shows a larger tumor (depicted by dark blue colour) in animals administered with intranasal PBS compared to a smaller tumor in the sCT-administered group. Confocal microscopy shows level of YAP (E), TAZ (F), pYAP/TAZ (G), LATS1 (H) pLATS1 (I) PKA (J) and pPKA (K) levels in the PBS and sCT-treated groups (Magnification-63x, scale bar-20 µm). Intensity refers to relative mean intensity. Data are presented as mean ± SD; *P < .05, **P < .01, ***P < .001, ****P < .0001.

Next, we investigated the ability of sCT to inhibit the growth of human and murine gliomas initiated by GSCs. Since sCT is a large peptide (32 amino acids) that cannot pass through the blood-brain barrier,^28^ we delivered sCT through the intranasal delivery route, an established route for the delivery of peptides and small molecules for the treatment of brain tumors and neurodegenerative diseases in mice.^29^^,^^30^ Intranasal delivery of sCT significantly inhibited the growth of glioma initiated by human (MGG4; Figure 4A, B, and D, *P* = .02) and murine (DBT-Luc; Supplementary Figure S7A, B, and D, *P* < .0001) GSCs and increased mouse survival (MGG4; Figure 4C, *P* = .02) and **(**DBT-Luc; Supplementary Figure S7C, *P* = .01). The sections derived from the MGG4-initiated small tumors formed in sCT-delivered mice showed reduced staining for total YAP and TAZ (Figure 4E and F; Supplementary Figure S7E and F), increased pYAP/pTAZ (Figure 4G), increased pLATS1 with no change in total LATS1 (Figure 4H and I), and increased pPKA with no change in total PKA (Figure 4J and K). From these results, we conclude that sCT inhibits GSC growth in vitro and glioma tumors initiated by GSCs through Hippo pathway activation in mouse models, thereby raising the possibility of using sCT for intranasal delivery in GBM therapy.

We next examined the distribution of sCT at the tumor site following intranasal delivery and the potential toxicities of sCT in mice. Miacalcin (synthetic salmon CT) is an FDA-approved drug for postmenopausal osteoporosis, as it inhibits osteoclasts.^31^ C57BL/6 mice bearing GSC-initiated tumors were delivered with two different concentrations of Cy3-labeled sCT through the intranasal route. Fluorescent imaging confirmed that Cy3-labeled sCT reached the tumor (Supplementary Figure S8A). Additionally, fluorescence imaging of various dissected organs was limited to the brain only (Supplementary Figure S8B). Toward assessing the possible toxicity, mice were administered varying amounts of sCT (2 IU, 4 IU, and 8 IU) intranasally daily for 4 weeks, and various parameters, including water consumption, food consumption, body weight, heart rate, ejection fraction, cardiac output, covariance of heart rate (CV%), hematological and biochemical parameters, were measured at the end of each week (Supplementary Figure S8C). We found no significant difference in all parameters in sCT-treated mice (Supplementary Figures S8D-J and S9A; Supplementary Table S3). H&E staining of the major organs obtained from the sCT-administered mice showed no histological alterations compared to those of control mice (Supplementary Figure S9B). From these results, we conclude that no adverse effects were observed in sCT-treated mice.

### MD Simulations of Patient-Derived CTR Mutants Elucidate the Mechanism behind Their Loss-of-Function

Our results thus far show that WT CTR activates the Hippo pathway to inhibit glioma cell proliferation. Tumor-derived mutations in CTR result in loss of function and fail to inhibit the growth of glioma cells.^11^ Out of the seven mutations, 3 (R45Q, A51T, and P100L) are on the extracellular domain (ECD), 2 (R404C and R420C) on the Helix-8 of intracellular domain (ICD), 1 (V250M) at the junction of transmembrane 3 (TM3) and intracellular loop 2 (ICL2), and one (A307V) on TM5 (Supplementary Figure S10A). As expected, CTR, with tumor-derived mutations, failed to activate the Hippo pathway, as evidenced by their inability to inhibit luciferase activity from the TEAD-Luc construct following sCT treatment (Supplementary Figure S10B). Additionally, we used CRE-Luciferase, a reporter plasmid to measure cAMP abundance.^32^ We found that the severe mutants A51T, V250M, and A307V also lost their ability to activate CRE-Luc activity compared to WT CTR upon sCT treatment (Supplementary Figure S10C), indicating impaired cAMP-mediated transcriptional signaling downstream of CTR activation. Collectively, based on the above experiments, we confirm that the patient-derived mutants have lost their function.

**Figure 5.**
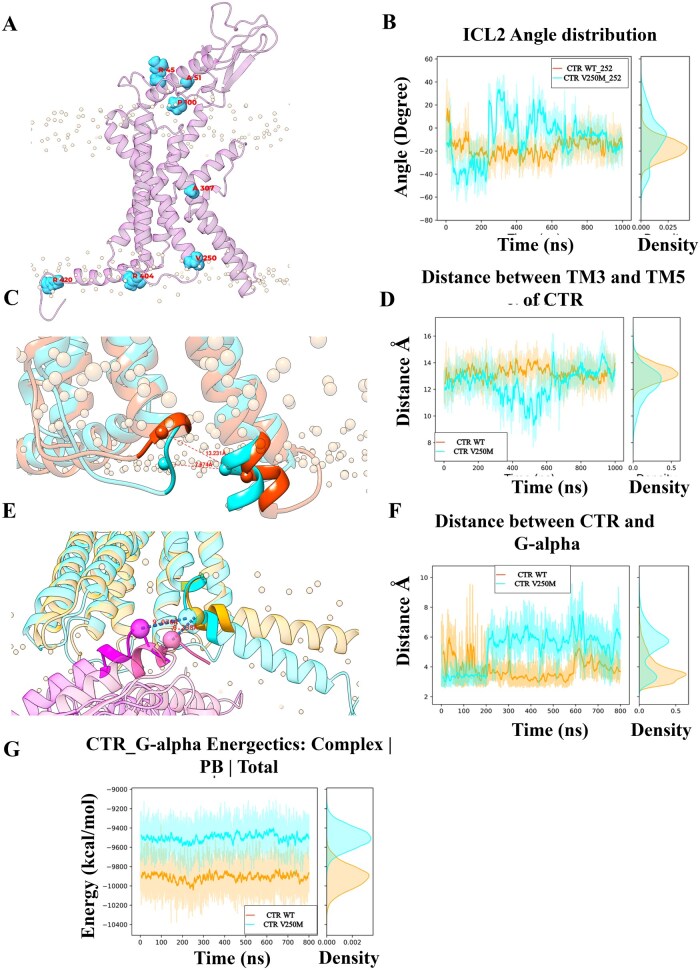
Illustration of mutant residue positions in CTR and differential conformation and interaction of V250M mutant compared to WT CTR. (A) Activated conformation of CTR with the position of seven patient-derived mutations is shown in cyan. (B) The plot shows ICL2 angle distribution in the WT CTR and V250M w.r.t. residue V252. (C) The figure shows the distance between the C-α carbon of the residue V/M250 in the transmembrane 3 (TM3) and residue K326 in the transmembrane-5 (TM5) in the WT CTR (orange) and V250M mutant (cyan). (D) The plot shows the distance between the Cα carbon of TM3 (V/M250) and TM5 (K326) between the WT CTR and the V250M mutant. (E) The plot shows the minimum distance between CTR (N396) and Gα (E392) in the WT CTR and V250M mutant. (F) The superimposed image shows the distance between the Cα carbon of residue N396 of CTR and residue E392 of Gα in the WT CTR (CTR in orange and Gα in pink) and V250M mutant (CTR in cyan and Gα in magenta). (G) Interaction energy between CTR and Gα in the WT CTR and V250M mutant. The red and blue color lines denote the WT CTR and V250M mutant, respectively.

To obtain insights into the effects of the patient-derived mutations on CTR function, we performed all-atom MD simulations of the WT and mutant CTR systems. A representative 3D schematic of the MD system, with the locations of the 7 mutants on the CTR marked, is shown (Figure 5A). All systems under consideration are listed (Supplementary Table S4). Using the WT system as a control, we compare simulation data across different mutant systems and quantify changes in 3D conformations and fluctuations to understand the impact of each mutation on CTR function.

#### V250M, an ICL2 mutant, affects ICL2 flexibility, TM5 conformation and Gα protein binding

Cryo-EM data (PDB ID: 6NIY) show that the 250th residue interacts with the G-protein.^13^ Hence, we examined the impact of V250M on the flexibility of ICL2 in MD simulations of activated WT and the V250M systems. To quantify ICL2 flexibility, we defined two planes: a primary plane using C-α atoms of residues from TM3 (G241 and I248) and TM4 (R260), and a secondary plane involving C-α atoms of TM3 (I248), TM4 (R260), and an ICL2 residue as the fourth (V252) (Supplementary Figure S10D). Trajectory data revealed that the angle distribution between these planes is broader in the V250M mutant than in the WT CTR (Figure 5B). Since the V252 site is in the loop region, we wanted to check whether the flexibility is local to the V252 residue or the overall loop. Hence, we quantified the angle distribution, keeping other residues on the loop as the fourth site (F253, T254, or E255 of ICL2). This analysis also revealed a broader angle distribution for all the residues tested (Supplementary Figure S10E-G), thereby establishing that, overall, the ICL2 conformational dynamics have increased due to the V250M mutation. These changes in loop dynamics are functionally important, as they can have a significant effect on the packing and conformation of proteins that could affect the protein-protein interaction network.^33-35^ Indeed, investigation of the conformational difference between the activated states of the WT CTR and V250M mutant revealed that the distance between the C-α atom of residue at the 250th position in TM3 and the C-α atom of residue K326 in TM5 was reduced to approximately 10 Å (closest ∼7 Å) for the V250M mutant in contrast to ∼13 Å observed consistently in the trajectory of the WT CTR (Figure 5C and D). Furthermore, we observed that in the V250M mutant, TM5 bends toward TM3 in the intracellular region, which is not observed in the WT CTR (Figure 5C). This conformational alteration is particularly significant because the intracellular segments of TM3 and TM5 are directly involved in interactions with Gα protein, suggesting that the V250M mutation could potentially impact the interaction between CTR and Gα protein.

We also investigated the impact of the observed conformational changes between WT CTR and V250M mutant on the interaction between Gα and CTR by measuring the distance between C-α atoms of specific residues of CTR (N396) and Gα (E392). We find this distance to be smaller in the WT CTR than in the V250M mutant, and the Gα is seen slightly shifted out of the binding site for the V250M mutant (Figure 5E and F). This suggests that the V250M mutant may have a weaker binding affinity for Gα than the WT CTR. Furthermore, our examination using MMPBSA-based interaction energy calculations^36^^,^^37^ showed that the interaction energy between CTR and Gα is lower in the WT CTR than in the V250M mutant (Figure 5G). This further corroborates that V250M mutation weakens the interaction between CTR and Gα and consequently could affect the downstream signaling.

#### C terminal Helix-8 mutants, R404C and R420C, show reduced membrane interactions

**Figure 6.**
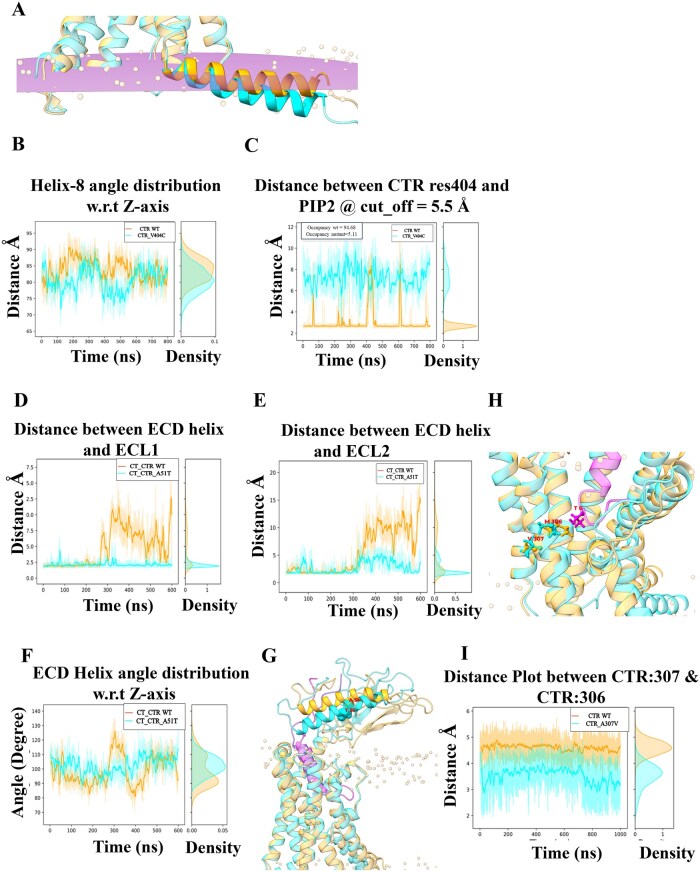
Conformational shift and differential interaction due to mutant CTRs R404C, A51T, and A307V with membrane lipid, ECLs, and CT, respectively, compared to WT CTR. (A) The superimposed image shows the orientation of the 8^th^ helix in the WT CTR (orange) and R404C mutant (cyan) CTR. (B) The plot shows the difference in the angle distribution of the 8^th^ helix with the z-axis in the WT CTR and R404C mutant. (C) The plot shows the interaction of the 404^th^ residue with PIP2 lipids of the membrane in the WT CTR and R404C mutant. (D**)** The superimposed image of WT CTR (orange) and A307V mutant CTR (cyan) shows the differential orientation between residues A/V307: CTR, M306: CTR, and T6: CT between WT CTR and mutant systems. (E) The plot shows the minimum distance between the side chain of residue A/V307 and M306 for WT CTR and the A307V mutant. (F) The plot shows the minimum distance between the ECD helix and ECL1 loop for WT CTR and A51T mutant when bound to CT. (G) This plot shows the minimum distance between the ECD helix and ECL1 loop for the previously mentioned systems. (H) The plot shows the ECD helix angle with respect to the Z-axis for the systems, the same as (F). (I) The superimposed image shows the ECD orientation between WT CTR (orange) and A51T mutant (cyan) when bound to CT (pink).

The C-terminal mutation R404C is located within the central segment of Helix-8, while R420C resides within the unstructured C-terminal region following Helix-8 (Figure 5A and Supplementary Figure S10A). To analyze the impact of these mutations, we calculated the angle between Helix-8 (the axis joining the C-α atoms of N396 and N414) and the membrane normal (Z-axis). We find that the Helix-8 moves away from the membrane plane for the R404C mutant (Figure 6A and B) as well as for the R420C mutant (Supplementary Figure S11A and B) as compared to WT CTR. We also measured the residue 404 occupancy with PIP2 lipids on the membrane and found that R404 of WT CTR interacts closely with the PIP2 lipid with an occupancy of 94.68% and an average proximity of 2.5-3.0 Å. On the other hand, the distance is increased to 6-8 Å in the R404C mutant with a significantly lower occupancy of 5.11% (Figure 6C). Similarly, the distance between residue 420 and the PIP2 lipid is approximately 4-6 Å in the R420C mutant with an occupancy of 62.16% compared to around 2.5-3 Å in the WT CTR with a 99.98% occupancy (Supplementary Figure S11C). We also show the molecular-scale interaction profile for the PIP2-Helix 8 complex from the bottom view and side view for the R404C mutant (Supplementary Figure S11D and 11E, respectively) and for the R420C (Supplementary Figure S11F and G, respectively). While the front view (Supplementary Figure S11A) revealed that the Helix-8 had swiveled away from the membrane with significantly reduced membrane contact, the bottom and side views clearly show the loss of interaction with the PIP lipids. As seen for the R404C mutant (Supplementary Figure S11D and E, respectively) and for R420C (Supplementary Figure S11F and G, respectively), in the wild type, the Arginine interacts with the PIP2 lipid, while the mutant Cysteine moves away from PIP2 lipids with its side chain facing outwards. It has been demonstrated both experimentally^38^ and through simulation studies^39^ that the interaction between anionic lipids, such as PIP2, and the basic residues of GPCRs promotes/stabilizes the active conformation of GPCRs, thereby strengthening the interaction between basic residues at the interface of GPCRs and Gα. An equivalent interaction is observed between Arginine residues at the 404th and 420th positions and PIP2 lipids, and this interaction is lost when the Arginines are mutated to Cysteines. Hence, the Arginine-to-Cysteine mutation at positions 404 or 420 is likely to weaken the CTR interaction with G-protein complexes, possibly affecting CTR activity.

#### ECD mutants, R45Q, A51T, and P100L, affects ECD and ECL1/ECL2 interaction

Next, we investigated the three ECD mutations, R45Q, A51T, and P100L (Figure 5A and Supplementary Figure S10A). All three mutants induce noticeable structural changes in CTR-ECD that could have functional implications. The ECD with a long alpha-helix (F39-Q60) assumes a lid-like feature in the CTR. Since ECD conformations dictate CT recruitment and binding, we decided to focus on how the ECD alpha-helix interfaces with the ECL1 (P207 to P216) or ECL2 (F285 to H296) of the TM region of the protein for the WT CTR and for the ECD mutants. To achieve this, we calculated the minimum distance between the ECD-helix and the ECLs. Also, much like the Helix-8, we aimed to characterize the helix orientation relative to the membrane normal. Analyses show that for the A51T mutant, the ECD closes in on the TM region of CTR (around 2.0-4.0 Å) and acts like a tight lid, whereas the ECD has an expansive breathing motion in the WT CTR (2.0-20.0 Å). Figure 6D and E shows the minimum distance distribution between ECD-ECL1 and ECD-ECL2, respectively, for both WT CTR and A51T mutant. Concomitantly, the WT CTR is also observed to have higher fluctuations in the helix angle than the A51T mutant (Figure 6F). Similar behavior, though to a lesser extent, is observed for R45Q (Supplementary Figure 12A-C) and P100L (Supplementary Figure S12D-F).

Distance and angle analysis of the ECD helix, as well as conformational dynamics insights from the trajectories (Figure 6G for A51T; Supplementary Figure S12G and H for R45Q and P100L, respectively), clearly reveal that the ECD is more dynamic and flexible and favors an open conformation for WT CTR in the ligand-bound state compared to the mutants. Another interesting insight from our simulation studies is that the ECD adopts a closed-lid state when the WT CTR is not bound to the CT ligand (data not shown). It appears that the binding of CT induces spatial rearrangements in the protein, disrupting the ECD-helix interactions with ECL1 and ECL2, leading to a highly breathable, open ECD. ECD mutants, on the other hand, don’t disrupt these cross-interactions. Even though we observe conformational rigidity of ECD-ECLs in mutants structurally, the origin of this change could be a result of a combination of multiple short- and long-range weak interactions, as it is not straightforward to directly single out a specific set of interactions that induce this change.

#### TM5 mutant, A307V, weakens the interaction between CT and CTR

CT binding to WT CTR induces conformational changes in CTR, with possible implications in downstream signaling.^7^ Next, we studied the impact of ECD-juxtaposed residue in TM5 (A307V) on CT binding to WT CTR. We found a subtle effect of mutation on the strength of CTR-CT binding. The sixth residue of CT (T6) interacts with residue M306 of WT CTR, and there is a weaker interaction between the side chains of A307 and M306 on TM5 (Figure 6H). When the A307V mutation occurs, due to the increased reach of Valine, the interaction strength between V307 and M306 increases as the minimum distance between their side chain decreases (Figure 6I). It is likely that this stronger interaction between V307 and M306 weakens the interaction between M306: CTR and T6: CT. Such hallmark subtle interactions are often widespread in GPCR systems,^40^ and high-resolution AAMD simulations often help unravel these networks, as shown above.

Together, molecular simulation data at atomic resolutions helped us understand the effect of mutants on the CTR signaling pathway from both structural and conformational dynamics perspectives. For example, our data show that a mutation in the intracellular region of the protein (V250M) significantly enhances ICL2 flexibility, which, in turn, can affect G-protein binding. We also found that R404C and R420C mutations in the intracellular region markedly reduce the interaction strength between helix-8 and the membrane, potentially impacting the active CTR population and its interaction with G-proteins. Our analyses of mutants located in the ECD (R45Q, A51T, and P100L) indicate that these mutants shift the ECD toward an alternate configuration, potentially influencing CT retention and binding. The A307V mutant on TM5 exhibits enhanced interaction with CT-binding residues, which may, in turn, weaken the CT-CTR signaling. In conjunction with available experimental data, our *in silico* conformational landscape investigations of the WT and mutant CTR systems (in the presence of CT and G-proteins) can be used to develop a hypothesis regarding the molecular mechanisms of CTR activation in health and disease states.

## Discussion

In our previous study, the neuroactive ligand-receptor interaction pathway emerged as one of the prominently mutated pathways with prognostic value for GBM patient survival. Notably, CTR was identified as the most frequently mutated gene, with mutations detected in 3.0% of GBM patients; such patients with mutated CTR had poor survival outcomes. Interestingly, sCT treatment of glioma cells with WT CTR inhibited the growth of glioma *in vitro* and *in vivo*.^11^ However, the underlying mechanism of this tumor suppressor function of the CT/CTR axis has remained elusive. In this study, we found that the sCT/CTR axis promotes the degradation of YAP/TAZ, well-known oncogenic transcription factors, by activating the Hippo pathway. Rescue experiments with the phosphorylation-resistant YAP (YAP S127A), which functionally overrides endogenous YAP activity, supported that YAP degradation is essential for sCT-mediated growth inhibition. Hippo pathway activation required cAMP/PKA/LATS1 signaling pathway. Intranasal delivery of sCT effectively inhibited glioma growth. Dynamis simulation studies revealed defects in the interactions between key residues in mutant CTR upon CT binding, unlike WT CTR.

The Hippo pathway, which is conserved across higher vertebrates, is a critical signaling pathway that regulates organ size and maintains tissue homeostasis.^2^ Dysregulation of the Hippo pathway is reported in multiple cancers, including GBM.^41^^,^^42^ Our investigation revealed that treatment of GBM cells with sCT inhibited YAP and TAZ through LATS1-dependent phosphorylation. We also found that the cAMP-PKA-LATS1 pathway mediated the regulation of YAP and TAZ by the CT/CTR axis. This regulatory axis aligns with findings in muscle stem cells, where the CTR-PKA-LATS axis similarly regulates YAP and TAZ activity, thereby contributing to the maintenance of muscle stem cells.

It is now well established that GSCs, which form small proportions of the tumors, are the tumor-initiating cells that drive tumor growth and impart resistance to conventional therapy^43^ underscoring the need to identify targets within the therapy-resistant pool of GSCs to develop add-on therapies that increase the efficacy of the current treatments. Interestingly, our investigation revealed elevated CTR expression in patient-derived GSCs compared with matched DGCs. We also found high levels of YAP and TAZ in patient-derived GSCs. Involvement of YAP and TAZ in preserving the stemness and plasticity of GSCs in GBM and other cancers.^44^^,^^45^ Thus, the high levels of CTR and YAP/TAZ observed in GSCs make them suitable candidates for sCT-based glioma therapy. Our results show that treatment of GSCs with sCT resulted in a pronounced inhibition of GSC growth. Furthermore, analysis of whole-tumor transcriptome data from TCGA RNA-seq, Affymetrix, and Agilent platforms revealed that CTR transcript levels are significantly higher in the mesenchymal gene expression subtype, but there is no difference in CTR transcript levels across G-CIMP, IDH mutation, and MGMT promoter methylation status in all 3 cohorts (Supplementary Figure S13). This suggests that mesenchymal GBM may be more suitable for sCT-based therapy. Further, intranasal administration of sCT resulted in an efficient reduction in tumor volume in both patient-derived human GSCs and murine GSCs in an intracranial orthotopic mouse glioma model. Collectively, these findings underscore the promising therapeutic potential of sCT in targeting tumor-initiating cell populations.

Structural and dynamic insights from microsecond-long simulation studies reveal possible activation mechanisms of CTR and provide a rationale for the loss of function in patient-derived mutants. Class B GPCRs, such as CTR, have a characteristically large extracellular domain that has evolved to detect and bind the ligands in a tightly regulated manner. Recent alanine-scanning mutagenesis data on ECL1^15^ and ECL2/ECL3^14^ from Sexton and coworkers clearly demonstrate how the ECL residues control the binding and signaling of distinct CTs. Interestingly, our data show that patient-derived mutant systems (R45Q, A51T, and P100L) exhibit a remarkably different orientational conformation of the ECD, with compromised ECL-ECD interactions that affect CT encapsulation. From this, we infer that CTR-ECD, through its interaction with ECLs, appears to act as a well-designed lid for CT recognition. Also, our data from the A307V mutation studies clearly reveal that CT-binding triggers a subtle packing rearrangement in the membrane-embedded TM region, which transfers the information to the G-protein binding ICL region of the CTR.^46-48^ Several reports in the GPCR literature document the crucial roles of ECLs^14^^,^^15^ and ICLs.^49^ V250M, a mutation on the ICL2 in CTR, causes profound changes in the ICL2 flexibility that affect the binding interface with the alpha-subunit of the G-proteins. Besides the altered interaction with CT and G-proteins, our simulation provides a structural origin of the loss of function by indicating a reduced interaction with the anionic lipids of the membrane and the CTR with the mutations, ie R404C and R420C, on Helix-8 and further down, respectively, a factor that could compromise the CTR function. Beyond structurally connecting the CT/CTR signaling axis and providing mechanistic insights into the signaling cascade, the conformational landscape presented by the simulation studies could also be used to rationally design more effective CT-mimics as therapeutic agents to restore the YAP-TAZ activity.

Overall, this study provides significant insights into the mechanism underlying the growth-inhibitory pathway of the CT/CTR axis in GBM, primarily by activating the Hippo signaling pathway. Furthermore, we have substantiated the therapeutic significance of intranasal sCT treatment in glioma. The salmon CT utilized in this research is an approved drug for women with post-menopausal osteoporosis, intended to maintain bone strength. Repurposing salmon CT for GBM patients with wild-type CTR holds promise for inhibiting glioma growth.

## Supplementary Material

vdag152_Supplementary_Data

## Data Availability

All data used in this work can be acquired from the corresponding author upon reasonable request.
