# Supplementary material for: Repurposing salmon calcitonin for glioblastoma treatment: Targeting Yes-associated Protein (YAP)/ Transcriptional co‑activator with PDZ‑binding motif (TAZ) via hippo pathway activation
Source: Neurooncol Adv. 2026 Jun 5;8(1):vdag152. doi: 10.1093/noajnl/vdag152 (PMC13326753; doi:10.1093/noajnl/vdag152)
Supplement: vdag152_Supplementary_Data [file vdag152_supplementary_data.zip › Supplementary Table 4.docx]

**Supplementary Table 4:** The various systems for AAMD simulations: A list of all systems with the overall system size in terms of the number of atoms and the total simulation time.

| # | System Name | #atoms | Time (ns) |  |
| --- | --- | --- | --- | --- |
| 1 | CALCR-WT | 390823 | 1000 | 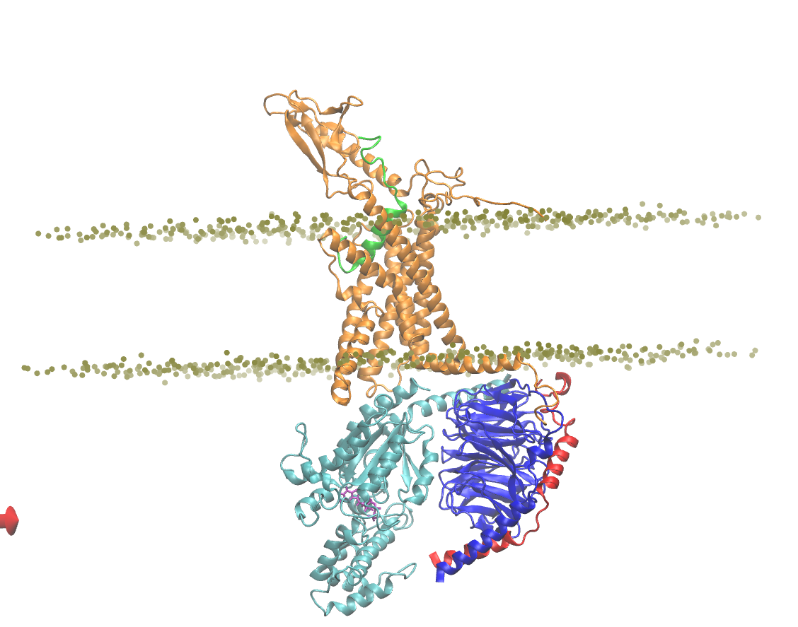  The figure shows full-length CALCR (Orange) bound to CT (Green) in the extracellular region. In the intracellular region, CALCR is bound to the G-protein complex consisting of Gα (Cyan), G-β (Blue), and G-γ (Red). The G-α is bound to the GDP molecule (Pink). Only the phosphate plane (Brown) of the membrane bilayer is shown here. The rest of the bilayer atoms, water, and ions are not shown for clarity. |
| 2 | CALCR-WT-truncated | 250540 | 800 |  |
| 3 | CALCR-R45Q | 271454 | 600 |  |
| 4 | CALCR-A51T | 270973 | 600 |  |
| 5 | CALCR-P100L | 337062 | 600 |  |
| 6 | CALCR-V250M | 373900 | 1000 |  |
| 7 | CALCR-A307V | 337369 | 1000 |  |
| 8 | CALCR-R404C | 373962 | 800 |  |
| 9 | CALCR-R420C | 375124 | 1000 |  |
| 10 | CT-CALCR-WT | 373962 | 800 |  |
| 11 | CT-CALCR-R45Q | 380292 | 600 |  |
| 12 | CT-CALCR-A51T | 377517 | 600 |  |
| 13 | CT-CALCR-P100L | 380162 | 600 |  |
| 14 | CALCR-V250M-G-GDP | 470099 | 800 |  |
| 15 | CALCR-WT-G-GDP | 571048 | 1000 |  |
| CT: Calcitonin  CALCR: Calcitonin Receptor  G-GDP: G-protein bound to GDP | | | |  |
