# Supplementary material for: Repurposing salmon calcitonin for glioblastoma treatment: Targeting Yes-associated Protein (YAP)/ Transcriptional co‑activator with PDZ‑binding motif (TAZ) via hippo pathway activation
Source: Neurooncol Adv. 2026 Jun 5;8(1):vdag152. doi: 10.1093/noajnl/vdag152 (PMC13326753; doi:10.1093/noajnl/vdag152)
Supplement: vdag152_Supplementary_Data [file vdag152_supplementary_data.zip › CTR manuscript -Supplementary files_Kumar (1) (1).doc]

**Supplementary file**

**Ms**: **Repurposing Salmon Calcitonin for Glioblastoma treatment: Targeting YAP/TAZ via Hippo pathway activation**

**Other Methods and Materials**

**Cell lines used**

We received the human tumor-derived GSCs MGG4 and MGG8 as generous gifts from Dr. Wakimoto at Massachusetts General Hospital, Boston, USA. The GB3 neurosphere was established in our lab using GBM tumor samples. The DBT-Luc neurospheres were provided by Dr. Dinesh Thotala at Washington University in St Louis, MO. The LN229 cell line was procured from ATCC (# CRL-2611, https://www.atcc.org/products/crl-2611), and the T98G cell line from ATCC (# CRL-1690, https://www.atcc.org/products/crl-1690). The U87 cell line was purchased from the EACC (#89081402). All the cell lines were procured from their respective sources within the last year, with periodic authentication procedures conducted as necessary. Mycoplasma contamination was routinely monitored and confirmed absent using the Ezdetect PCR Kit for Mycoplasma Detection (HiMedia).

**Neurosphere culturing**

GSCs were isolated from GBM tumor tissue following a series of steps. Initially, the tissue was dissected and treated with trypsin, followed by the addition of a trypsin inhibitor. The treated tissue was mechanically dissociated and filtered to remove any debris. The resulting filtrate was cultured in ultra-low attachment plates in a specialized stem cell medium suitable for neurosphere formation. These neurospheres were cultured in a Neurobasal medium supplemented with various growth factors and supplements, including l-glutamine, heparin, B27 supplement, N2 supplement, rhEGF, rhFGF-basic, and antibiotics. After seven days of culture, the neurospheres were chemically dissociated into single-cell suspensions using NeuroCult Chemical Dissociation Kit for subsequent replating.

**Differentiation of GSCs to DGCs**

To induce differentiation of GSCs into DGCs, mature neurospheres were harvested from ultra-low attachment plates (Corning, USA). These neurospheres were transferred onto standard cell culture dishes and cultured in Dulbecco's Modified Eagle Medium (DMEM) supplemented with 10% fetal bovine serum (FBS) and antibiotics, as previously described, for 15–20 days, with medium replacement every 3–4 days. Differentiation was confirmed by morphological changes and reduced expression of stem cell markers, along with increased expression of differentiation markers^1^.

**Lentivirus preparation and transduction of cells**

HEK-293T cells were seeded onto poly-l-lysine-coated cell culture dishes of either 60 mm or 90 mm diameter. Upon reaching 60–70% confluency, the cells were transfected with shRNA plasmid alongside helper plasmids psPAX2 and pMD2.G using Opti-MEM medium (Invitrogen #22600-050) and Lipofectamine 2000 (https://www.thermofisher.com/order/catalog/ product/12566014; Invitrogen) facilitated the transfection process. Following a 6-hour incubation period post-transfection, the Opti-MEM medium was replaced with fresh DMEM supplemented with 10% FBS. After 60 hours, the supernatant containing the transfected cells contents was harvested into 15 ml Falcon tubes, subjected to centrifugation at 5000 rpm for 10 minutes, filtered through a 0.45 μm filter, and finally stored at –80°C for future applications. The shRNA clones used in the study were taken from the Sigma human TRC shRNA library. For the knockdown of CTR, the shRNA clones used were TRCN0000008162 and TRCN0000008163. The shRNA clones TRCN0000196268 and TRCN0000195739 were used for the knockdown of LATS1.

**Luciferase and beta-galactosidase (β-gal) assay**

Cells were seeded into six-well plates at approximately 60% confluency and co-transfected with a luciferase reporter construct (3µg) and pCMV-beta gal (1 µg, as a transfection control) with the help of Lipofectamine 2000 reagent following the manufacturer's instructions. After 24 hours of transfection, cells were treated with sCT for 4 hours before being harvested. Cells were lysed using the reporter lysis buffer (Promega), and the protein concentrations were estimated using the Bradford assay reagent (Bio-Rad). Subsequently, 10 µg of protein was added to 50 µl of luciferase assay reagent (LAR) to evaluate luciferase activity using a luminometer. β-galactosidase activity was measured from the same lysates using a colorimetric assay, and luciferase activity was normalized to β-galactosidase activity to account for differences in transfection efficiency.

**RNA isolation, cDNA conversion, and Real-Time quantitative qPCR**

Total RNA from cells was isolated using TRI reagent following the manufacturer's protocol. Subsequently, the RNA quality was determined by electrophoresis on a 2% MOPS-formaldehyde gel, and RNA was quantified using NanoDrop. 2 µg of total RNA was used for cDNA conversion using a high-capacity cDNA reverse transcription kit. The cDNA was diluted to a final concentration of 10 ng/µl. Real-time quantitative PCR was carried out using ABI Quant Studio 5 and 6 instruments, using the DyNAmo Flash SYBR Green qPCR kit. Gene-specific primer sets were used under defined cycling conditions for the reaction. Housekeeping genes such as GAPDH, ACTB, 18S rRNA, RPL35a, and ATP5G were used as human gene expression analysis references. Gene expression was calculated using the ΔΔCT method, transformed to a log2 ratio, and converted to the absolute scale for visualization. The primers used for RT-qPCR are shown below.

List of primers used in the study

| **Gene** | **Forward Primer (5’-3')** | **Reverse Primer (5’-3’)** |
| --- | --- | --- |
| **YAP1** | TGTCCCAGATGAACGTCACAGC | TGGTGGCTGTTTCACTGGAGCA |
| **TAZ** | GAGGACTTCCTCAGCAATGTGG | CGTTTGTTCCTGGAAGACAGTCA |
| **CTGF** | CTTGCGAAGCTGACCTGGAAGA | CCGTCGGTACATACTCCACAGA |
| **CYR61** | GGAAAAGGCAGCTCACTGAAGC | GGAGATACCAGTTCCACAGGTC |
| **CTR** | CCTTTTCAAATCAAACCTATCCAAC | GGACAATACTCCAGCCGGT |
| **LATS1** | CACTGGCTTCAGATGGACACAC | GGCTTCAGTCTGTCTCCACATC |
| **SOX2** | AACCCCAAGATGCACAACTC | GCTTAGCCTCGTCGATGAAC |
| **SALL2** | TAATCTCGGACTGCGAAGGT | TAGAACATGCGTTCTGGTGG |
| **OLIG2** | CCAGAGCCCGATGACCTTTT | AGGACGACTTGAAGCCACTG |
| **POU3F2** | TGACGATCTCCACGCAGTAG | GGCAGAAAGCTGTCCAAGTC |
| **GAPDH** | TTGTCAAGCTCATTTCCTGG | TGATGGTACATGACAAGGTGC |
| **RPL35** | AGCTCTCTAAGATCCGAGTCG | GAACACGGGCAATGGATTTCC |
| **18S** | GCAGAATCCACGCCAGTACAAG | GCTTGTTGTCCAGACCATTGGC |
| **ATP5G** | CCAGACGGGAGTTCCAGAC | GACGGGTTCCTGGCATAGC |

**Western Blotting**

For Western blot, cell pellets were lysed in RIPA lysis buffer containing specific protease and phosphatase inhibitors. Following lysis, the cells were centrifuged, and the protein supernatant was collected. The protein concentration in the sample was estimated using Bradford's reagent, with a BSA curve serving as a standard reference. Equal protein amounts were added with loading dye, denatured at 90°C for ten minutes, and loaded onto an SDS PAGE gel (8-10%), and resolved using stacking and resolving gels of appropriate concentrations. Proteins were then transferred onto PVDF membranes using a semi-dry transfer apparatus. After blocking with skimmed milk, the membrane was probed with primary antibodies overnight at 4°C, followed by washing three times and incubation with secondary antibodies. Subsequently, the blot was visualized using chemiluminescent reagents and imaged using a BIO-RAD chemidoc imaging system.

**Antibodies and reagents used**

The primary antibodies used in the study were purchased from the following companies: CTR (Invitrogen PA5-25594, 1:1000), YAP (CST 14074S, 1:1000), TAZ (CST 4883S, 1:1000), pYAP (CST 4911S, 1:1000), CTGF (CST 86641S, 1:1000), LATS1 (CST 9153S, 1:500), pLATS1 (CST 8654S 1:500), MST1 (CST 3682S, 1:1000), pMST1 (CST 49332S), GFP (CST 2956S), PKA (CST4782S, 1:1000), pPKA (CST 5661S, 1:1000), SOX2 (Abcam ab92494, 1:2000), GAPDH (SIGMA G8795, 1:25000). Goat anti-rabbit IgG (H+L) secondary HRP conjugate (Invitrogen# 31460, WB 1:5000), Goat anti-mouse IgG (H+L) secondary HRP conjugate (Invitrogen# 31430, WB 1:5000), goat anti-rabbit IgG (H+L) highly cross-adsorbed secondary antibody, Alexa Fluor 488 (Invitrogen, Cat# A-11034), goat anti-rabbit IgG (H+L) highly cross-adsorbed secondary antibody, Alexa Fluor 594 (Invitrogen, Cat# A-11037). All the Alexa Fluor antibodies were diluted at 1:500 for ICC and IHC. Salmon calcitonin (Unicalcin, 100 IU) was obtained from a local pharmacy. The list of small-molecule inhibitors used in this study is given below.

**List of small molecules** used in the study

| **S.No.** | **Inhibitor name** | **Target molecule** | **Catalog Number** |
| --- | --- | --- | --- |
| **1.** | TRULI | LATS | HY138489 (Medchem Express) |
| **2.** | H89 | PKA | B1427 (Sigma Aldrich) |
| **3.** | Forskolin | Adenylyl cyclase | F6886 (Sigma Aldrich) |

**Apoptosis**

The Annexin V Apoptosis Detection Kit from BD Biosciences was used to quantify apoptotic cells. Followed by sCT treatment (300 nM) for 72 hours, cells were washed twice with cold PBS and then suspended in a binding buffer at a concentration of 1 × 10^6 cells per ml. Subsequently, 100 μl of this suspension (equivalent to 1 × 10^5 cells) was transferred to a 5-ml culture tube, followed by the addition of Annexin V and propidium iodide. Following a 15-minute incubation at room temperature in the dark, an additional 400 μl of binding buffer was added to each tube. The cells were then analyzed using a flow cytometer (FACS Verse, BD Biosciences) within 1 hour of staining.

**Colony Formation**

LN229 cells were seeded at a density of 1x10^3 cells per well in a 6-well plate. After 24 hours, the cells were subjected to treatments with either PBS or sCT (100 nM), which were subsequently replenished every other day for 10 days by partial medium replacement. On the 11th day, the cells were fixed using chilled methanol, stained using crystal violet, and imaged. Utilizing the ImageJ software, the quantification of both the percentage and intensity of crystal violet-stained cell colonies covering the area was conducted.

**Cell Viability**

LN229 cells were seeded at a density of 1.0 × 10^3 cells per well in a 96-well plate. Following seeding, the cells were treated with a specific concentration of sCT (nM) for 72 hours. After the treatment period, MTT reagent was added to each well and incubated for 3 hours to allow the formation of Formazan crystals. Following incubation, DMSO was used to dissolve the Formazan crystals, and the absorbance was measured at 570 nm. The obtained absorbance values were used to generate a line graph representing the cell viability data.

**Intracranial injection of GBM cells**

Cells were collected using incomplete NBM or F12 media, depending on the specific cell type intended for injection. 0.1 × 10^6 DBT-Luc GSCs, and 0.15x10^6 MGG4 Luc GSCs were intracranially injected as a 5ul cell suspension into each animal's hippocampus at a depth of 2.5 mm using a stereotaxic apparatus. All procedures were carried out under anesthesia, and animals received appropriate post-operative care. Imaging of the animals commenced on the fifth day post-injection and continued every 5–6 days until the experiment's conclusion. sCT treatment began seven days post-injection, with animals receiving a daily intranasal dose of 4 IU sCT (2 IU in the morning and 2 IU in the evening) every day for the next 25 days. Intranasal drug delivery was performed following the protocol mentioned earlier^2^.

**In vivo Imaging**

In vivo bioluminescence imaging was performed using the PerkinElmer IVIS Spectrum system under mild gas anesthesia with isoflurane. Prior to imaging, mice were administered D-luciferin, and bioluminescent signals were acquired and quantified using the IVIS imaging software.

**Haematoxylin and Eosin staining**

Following perfusion with 4% PFA, mouse brains were embedded in paraffin blocks and sectioned into 5 μm slices using the Leica Biosystems microtome instrument. These sections were affixed onto glass slides, deparaffinized, rehydrated, and subsequently stained with Harris Hematoxylin (Merck, 6159380051046) for nuclear visualization and Eosin Y (SDFCL, 44027G25) for cytoplasmic staining. After staining, the sections were mounted using DPX mounting medium and imaged at 0.8× magnification using a Lawrence and Mayo digital microscope.

**Cy3 Labelling of Calcitonin Peptide**

Labelling of Calcitonin Peptide is done according to the manufacturer's protocol. Briefly, Cy3 NHS Ester (Cytiva PA13101) and sCT (MedChemExpress HY‐P0090**)** were incubated together overnight at 4°C with 1x PBS (pH=7.2) and 1mM of Sodium bicarbonate in a 5:1 mM ratio. After overnight incubation, the labelled peptide is purified through reverse-phase HPLC.

**Intranasal delivery of labelled peptide**

Tumour-bearing nude mice were administered PBS, unlabelled calcitonin (10 µg), and Cy3-labelled calcitonin (5 µg & 10 µg) via intranasal delivery. After 3 hours of administration, mice underwent whole-body fluorescence imaging with a 528nm excitation filter via the IVIS system. Immediately after whole body fluorescence imaging, all the mice were anaesthetised using isoflurane, and major organs (Brain, Lungs, Heart, kidneys, Liver, and Spleen) were harvested and subjected to organ-specific fluorescence imaging using the same excitation filter.

**Sub-acute toxicity of calcitonin in C57BL/6 mice**

C57BL/6 (6 weeks of age) were administered PBS or sCT at doses of 1 I.U., 2 I.U., or 4 I.U. via intranasal delivery, morning and evening daily, for a consecutive 28 days. To eliminate sex differences, only female mice were used in the toxicity studies. Body weight, ECG recording, food consumption, and water consumption were measured every 7 days during the treatment. Blood samples and tissues were collected at 24 hrs following the final sCT/PBS treatment. Blood samples for haematological analyses were collected in Microtainer Blood Collection Tubes with K2EDTA. Peripheral blood was analysed, and the haematological and biochemical parameters were analysed. Major organs, including the brain, lung, heart, liver, spleen, and kidney, were harvested from mice after the completion of sCT/PBS treatment. Histological slides prepared from these organs were independently analysed by pathologists.

**Survival Analysis**

The survival analysis employing the Kaplan-Meier method was done using GraphPad Prism software (GraphPad Software, San Diego, CA).

**Quantification and Statistical Analysis**

Bar graphs and Kaplan–Meier survival curves were generated using GraphPad Prism software. Statistical analyses were performed using unpaired Student’s *t*-tests or one-way/two-way ANOVA, as appropriate. All *p*-values were calculated using GraphPad Prism. The significance of the Kaplan-Meier survival analysis was determined via the log-rank test. Pearson's correlation coefficient was employed to calculate correlation coefficients using GraphPad Software. Experimental replicates, except those involving animal models, were conducted at least three times with duplicates for each experimental condition. Data are presented as mean ± standard deviation. A p-value <0.05 was considered significant, denoted by *, **, ***, and **** for p-values <0.05, 0.01, 0.001, and 0.0001, respectively, while "ns" indicates insignificant.

**Molecular Modelling of CTR:**

**Insilico reconstitution of full-length CTR from structurally solved fragments**

For our simulation study, we reconstituted the full-length CTR using the available structures of the transmembrane region of the CTR (PDB ID: 6NIY)^3^ and the solved CT-bound extracellular domain (PDB ID:5IIO^4^). Both of the above-mentioned PDBs are taken as the template, and the full-length CTR was modeled using the MODELLER tool^5^. In the PDB: 5II0, the ECD of CTR is bound to the unstructured region of the C-terminus of CT, while in another cryo-EM solved structure (PDB:6NIY), the TM region of CTR is bound to the helical N-terminal region of CT. By carefully superimposing both structures onto our model, we were able to find the exact position and orientation of full-length functional CT when it's bound to the full-length functional CTR. While reconstructing the ECD for molecular simulations, we have included the disulfide bonds as per the available experimental information^3^. Also, we have deleted the first 24 residues that make up the protein's signal peptide region. We have modeled the CTR till residue number 428, as a recent study from the Patrick Sexton (Monash University, Australia) group exhibited that the CTR was fully functional with the deletion of the C-terminal till residue 428^3^. We chose the membrane composition to mimic the astrocyte membrane. The following composition was used: POPC:CHOL:SOPS:POPE:PSM:PIP2: 48%:20%:10%:10%:7%:5%.

**Insilico reconstitution of mutant CTR with binding partners**

Along with the WT, we also modeled seven different mutant states of the protein. Three of these single point mutations (R45Q, A51T, P100L) are in the ECD domain, A307 is in TM5, V250M mutant is located on the ICL2, and the last two mutants (R404C, R420C) are in the amphipathic TM8. Besides constructing the seven mutant and WT systems for the CTR that act as our reference for the mutant states, we have also constructed separate CTR systems in complex with CT and G-proteins, both for the WT and mutant systems, based on the requirements of our study. For example, we have CT-bound ECD-mutant systems along with the CT-bound WT, where the WT CT-bound system acts as a control for changes observed in CT-binding due to mutations. Similarly, we have created WT CTR and mutant V250M in complex with the G-proteins since ICL2, which carries the amino acid at the 250^th^ position, is in contact with the G-protein.

**All-atom molecular dynamics simulation details:**

All the systems are built using the CHARMM-GUI input generator^6-8^. The CTR was inserted in a membrane size of 15 nm x 15 nm with the above-mentioned lipid composition. The protein-bilayer system was then solvated with the TIP3P water model with roughly 22 Å water padding. The full system was charge-neutralised with 150 mM of Na+ and Cl- ions.

The CHARMM36m forcefield was used as a particle definition and its interaction parameters^9^, and the GROMACS engine was used as the molecular simulation software^10-12^. The minimisation step was performed by the steepest descent algorithm, along with H-bond constrained using the LINCS algorithm. Then, two stages of NVT equilibration were performed with the Berendsen thermostat at a temperature of 303.15 K. Afterwards, four stages of NPT equilibration were done with the Berendsen barostat at 1 atm pressure. The further production run was extended upto 600 ns to 1 μs with a time step of 2 fs for different systems of CTR with Nose-Hoover thermostat at 303.15 K and Parrinello-Rahman barostat at 1 atm.

All the analyses were carried out using the MDanalysis modules^13, 14^**,** and the molecular figures were rendered with the help of the ChimeraX tool^15, 16^**.** For the binding energy calculation between CTR and Gα, the gmxMMPBSA tool was used^17, 18^**.** All our data, including the simulated trajectories and input files for our AAMD simulations for all the systems under consideration, are publicly available on our GitHub repository: <https://github.com/codesrivastavalab/GPCR-CalcitoninReceptor-AAMD>. The large trajectories are deposited on a publicly available SharePoint location, and a link to the location is available in the above GitHub repository.

**References**

1.	Suva ML, Rheinbay E, Gillespie SM, et al. Reconstructing and reprogramming the tumor-propagating potential of glioblastoma stem-like cells. Cell 2014;157:580–594.

2.	Eyme KM, Carvalho L, Badr CE. Intranasal delivery of experimental compounds in orthotopic brain tumor mouse models. STAR Protoc 2021;2:100290.

3.	Liang YL, Khoshouei M, Radjainia M, et al. Phase-plate cryo-EM structure of a class B GPCR-G-protein complex. Nature 2017;546:118–123.

4.	Johansson E, Hansen JL, Hansen AM, et al. Type II Turn of Receptor-bound Salmon Calcitonin Revealed by X-ray Crystallography. The Journal of biological chemistry 2016;291:13689–13698.

5.	Sali A, Blundell TL. Comparative protein modelling by satisfaction of spatial restraints. Journal of molecular biology 1993;234:779–815.

6.	Jo S, Kim T, Iyer VG, Im W. CHARMM-GUI: a web-based graphical user interface for CHARMM. Journal of computational chemistry 2008;29:1859–1865.

7.	Brooks BR, Brooks CL, 3rd, Mackerell AD, Jr., et al. CHARMM: the biomolecular simulation program. Journal of computational chemistry 2009;30:1545–1614.

8.	Lee J, Cheng X, Swails JM, et al. CHARMM-GUI Input Generator for NAMD, GROMACS, AMBER, OpenMM, and CHARMM/OpenMM Simulations Using the CHARMM36 Additive Force Field. Journal of chemical theory and computation 2016;12:405–413.

9.	Huang J, Rauscher S, Nawrocki G, et al. CHARMM36m: an improved force field for folded and intrinsically disordered proteins. Nat Methods 2017;14:71–73.

10.	Páll S, Abraham MJ, Kutzner C, Hess B, Lindahl E. Tackling Exascale Software Challenges in Molecular Dynamics Simulations with GROMACS. In: Markidis S, Laure E, ed. Solving Software Challenges for Exascale; 2015 2015//; Cham: Springer International Publishing: 3–27.

11.	Abraham MJ, Murtola T, Schulz R, et al. GROMACS: High performance molecular simulations through multi-level parallelism from laptops to supercomputers. SoftwareX 2015;1-2:19–25.

12.	Bekker H, Berendsen HJC, Dijkstra E, et al. GROMACS - A PARALLEL COMPUTER FOR MOLECULAR-DYNAMICS SIMULATIONS. 1993.

13.	Michaud-Agrawal N, Denning EJ, Woolf TB, Beckstein O. MDAnalysis: a toolkit for the analysis of molecular dynamics simulations. J Comput Chem 2011;32:2319–2327.

14.	Gowers R, Linke M, Barnoud J, et al. MDAnalysis: A Python Package for the Rapid Analysis of Molecular Dynamics Simulations. 2016:98–105.

15.	Goddard TD, Huang CC, Meng EC, et al. UCSF ChimeraX: Meeting modern challenges in visualization and analysis. Protein science : a publication of the Protein Society 2018;27:14–25.

16.	Meng EC, Goddard TD, Pettersen EF, et al. UCSF ChimeraX: Tools for structure building and analysis. Protein science : a publication of the Protein Society 2023;32:e4792.

17.	Miller BR, 3rd, McGee TD, Jr., Swails JM, Homeyer N, Gohlke H, Roitberg AE. MMPBSA.py: An Efficient Program for End-State Free Energy Calculations. Journal of chemical theory and computation 2012;8:3314–3321.

18.	Valdes-Tresanco MS, Valdes-Tresanco ME, Valiente PA, Moreno E. gmx_MMPBSA: A New Tool to Perform End-State Free Energy Calculations with GROMACS. Journal of chemical theory and computation 2021;17:6281–6291.

**Supplementary Figure Legends**

**Supplementary Figure 1**

**Inhibition of YAP and TAZ by sCT in T98G cell line**

Effect of PBS or sCT treatment on luciferase reporter activity in GBM cell lines. **(A–B)** LN229 cells transfected with TEAD-Luc **(A)** or CTGF-Luc **(B)** constructs were treated with PBS or sCT, and luciferase activity was measured (t-test; n = 3 per group for **A,** n = 4 per group for B). **(C–D)** T98G cells transfected with TEAD-Luc **(C)** or CTGF-Luc **(D)** constructs were similarly treated, and reporter activity was assessed. **(E)** RT-qPCR analysis shows YAP, TAZ, CTGF, and CYR61 transcript levels upon sCT treatment (300 nM) in the T98G cell line (two-way ANOVA; n=3/group) at the indicated time points. (**F)** Western blotting shows levels of YAP, TAZ, pYAP, pTAZ, and CTGF upon sCT treatment (300 nM) in the T98G cell line at the indicated time points. **(G)** Confocal microscopy analysis shows YAP and TAZ levels in LN229 cells with and without sCT (300 nM) after 4 hours of treatment. Red indicates YAP, green indicates TAZ, and blue indicates DAPI. The merged images are shown for representation. Magnification x20, scale bar 20 µm. The quantification of nuclear YAP and TAZ intensity is shown in the corresponding graph. Intensity refers to relative mean intensity. (**H)** Confocal microscopy analysis shows YAP and TAZ levels in T98G cells with and without sCT treatment (300 nM) after 4 hours. Red indicates YAP, green indicates TAZ, and blue indicates DAPI. The merged images are shown for representation. Magnification x63, scale bar 20 μm. The quantification of nuclear YAP and TAZ intensity is shown in the corresponding graph. Intensity refers to relative mean intensity. (**I)** MTT assay showing the viability of T98G cells at the indicated concentrations of sCT (nM) after 72 hours of treatment (one-way ANOVA; n=4/group). **p* < .05, ***p* < .01, ****p* < .001, *****p* < .0001.

**Supplementary Figure 2**

**Effect of sCT is CTR-dependent. (A)** Western blot showing the knockdown of CTR in LN229 cells using two shRNAs. (**B)** MTT assay showing the effect of sCT on LN229/ shNT and LN229/ shCTR cells at the indicated concentrations of sCT (nM) (two-way ANOVA; n=4/group); n=3/group). (**C)** Quantification of growth inhibition by sCT (100 nM) in shNT/LN229 and shCTR/LN229 conditions as seen in the colony formation assay (t-test; n=2/group). (**D)** TEAD Luc activity inhibition in shNT and shCTR knockdown conditions upon sCT treatment (300 nM) (t test; n=6/group). (**E)** Western blotting shows YAP, TAZ, pYAP, and pTAZ levels upon sCT treatment (300 nM) under shNT and shCTR conditions at the indicated time points. (**F)** TEAD Luc activity in LN229 cells pre-treated with DMSO or BAPTA-AM (10 µM) for 2 hours followed by sCT treatment (300 nM). (**G)** Confocal microscopy analysis shows YAP and TAZ levels in shNT and shLATS1 conditions with and without sCT treatment (300 nM) after 4 hours. Red indicates YAP, green indicates TAZ, and blue indicates DAPI. The merged images are shown for representation. Magnification x63, scale bar 20 µm. (**H)** MTT Assay revealing Cell Viability in shNT and shLATS1 Cells following sCT treatment at the indicated concentrations (two-way ANOVA; n=4/group) after 72 hours. Data are presented as mean ± SD; **p* < .05, ***p* < .01, ****p* < .001, *****p* < .0001.

**Supplementary Figure 3**

**sCT modulates YAP and TAZ in a cAMP-PKA-LATS1-dependent manner.** (**A)** LN229 cells were first transfected with the TEAD Luc construct and pre-treated with TRULI (10 µM) for 2 hours, followed by sCT treatment (300 nM) for 4 hours before cell harvesting for luciferase activity measurement (two-way ANOVA; n=6/group). (**B)** LN229 cells were pre-treated with TRULI for 2 hours and treated with sCT (300 nM) for the indicated time points. The lysates were assessed for total and phosphorylated YAP and TAZ. (**C)** Confocal imaging displays YAP and TAZ levels after sCT treatment (300 nM), with DMSO or TRULI (10 µM) pre-treatment. (**D)** Cell viability of LN229 cells was assessed by MTT assay upon sCT treatment at the indicated concentrations with and without TRULI pre-treatment (two-way ANOVA; n=3/group). (**E)** Confocal imaging shows YAP's abundance and nuclear localisation upon sCT treatment in DMSO or H89-pre-treated (10 µM) LN229 cells. (**F)** Cell viability of LN229 cells was assessed by MTT assay upon sCT treatment (nM) in DMSO or H89-pretreated (10 µM) LN229 cells (two-way ANOVA; n=4/group). (**G)** Levels of cAMP abundance at the indicated time points after sCT treatment (300 nM) were determined using the ELISA assay kit (t test; n=2/group). Data are presented as mean ± SD; **p* < .05, ***p* < .01, ****p* < .001, *****p* < .0001.

**Supplementary Figure 4**

**FSK mimics the effect of sCT**

(**A)** TEAD luciferase activity in LN229 cells treated with DMSO or FSK (10 µM) for 4 h (t test; n = 4/group). (**B)** Expression of YAP, TAZ, CTGF, and CYR61 upon FSK treatment (10 µM) at the indicated time points (two-way ANOVA n=3/group). (**C)** Western blotting shows the levels of YAP and TAZ and their phosphorylated forms upon FSK treatment (10 µM) at the indicated time points. (**D)** Confocal images depicting the expression levels of YAP and TAZ upon FSK treatment (10 µM). Red indicates YAP, green indicates TAZ, and blue indicates DAPI. The merged images are shown for representation. Magnification x 63, scale bar=20 µm. (**E)** Cell viability of LN229 cells with the indicated concentration of FSK treatment (10 µM) after 72 hours of treatment (t test; n=4/group). Data are presented as mean ± SD; **p* < .05, ***p* < .01, ****p* < .001, *****p* < .0001.

**Supplementary Figure 5**

**GSCs are better suited for sCT-mediated growth inhibition. (A).** qRT-PCR confirms the GSC and DGC status based on the levels of reprogramming factors SOX2, SALL2, and POU3F2 (t test; n=2/group). **(B)** TEAD-Luc activity in GB-3 neurospheres treated with PBS or sCT (300 nM) (t test; n=3/group). **(C)** mRNA expression levels of YAP, TAZ, CTGF, and CYR61 in GB3 cells treated with sCT (300 nM) (n=3/group). **(D)** Western blotting shows the levels of YAP, TAZ, pYAP, pTAZ, pLATS1, pPKA, and PKA in GB3 spheres treated with sCT (300 nM) at the indicated time points. **E, F, G** shows the impact of sCT (300 nM) upon the growth of GB3 neurospheres by sphere formation assay and limiting dilution assay, respectively. **(H)** MTT assay shows the differential growth inhibition of sCT on MGG4 GSCs vs DGCs (two-way ANOVA; n=4/group). Data are presented as mean ± SD; **p* < .05, ***p* < .01, ****p* < .001, *****p* < .0001

**Supplementary Figure 6**

**sCT inhibits the growth of human and murine GSCs. (A)** Western blot shows the levels of YAP, TAZ, pYAP and pTAZ upon sCT treatment (300 nM) at the indicated time-points. Impact of sCT (300 nM) on human-derived GSCs, MGG8, as shown by sphere formation assay **(B, C)** and limiting dilution Assay **(D). (E)** Western blotting shows the levels of YAP, TAZ, pYAP, pTAZ, PKA, and pPKA in murine GSCs, DBT-Luc. Impact of sCT (300 nM) on human-derived GSCs, MGG8, as shown by sphere formation assay **(E, F)** and limiting dilution Assay **(G)**. Data are presented as mean ± SD; **p* < .05, ***p* < .01, ****p* < .001, *****p* < .0001

**Supplementary Figure 7**

**Intranasal sCT administration inhibits DBT-Luc GSC-derived tumor in C57BL/6 mice. (A)** 0.1x10^6^ DBT-Luc GSCs were injected intracranially into the brain of C57BL/6 mice (n=5 per group). The tumor was allowed to grow for seven days, after which sCT was administered intranasally at a dose of 2 IU/ animal twice daily. In vivo, bioluminescence imaging of the animals was taken every five days from the date of injection. (**B)** The average radiance efficiency between the PBS and sCT-administered groups is plotted (two-way ANOVA; n=5/group). (**C)** The Kaplan-Meier graph shows the survival difference between the two groups of mice that were administered PBS or sCT (n=5/group). (**D)** Haematoxylin and eosin staining shows a larger tumor (depicted by dark blue colour) in animals administered with intranasal PBS compared to a smaller tumor in the sCT-administered group. Confocal microscopy shows YAP **(E)** and TAZ **(F)** levels in the PBS and sCT-treated groups. Intensity refers to relative mean intensity. Data are presented as mean ± SD; **p* < .05, ***p* < .01, ****p* < .001, *****p* < .0001

**Supplementary Figure 8**

**Bioavailability of sCT in the brain upon intranasal administration and toxicity profile of sCT in animals.**

**(A)** In vivo imaging shows fluorescence activity in the brain of animals after 3 hours of intranasal Cy3-tagged sCT-treatment. **(B)** Fluorescence activity in the major organs of the animals. **(C)** The illustration depicts the strategy to study the sub-acute toxicity of sCT in animals. Measures of **(D),** water consumption **(E)**, Food consumption **(F),** body weight, **(G)** heartbeat (beat per minute) **(H)**, ejection fraction (%) **(I)**, cardiac output (ml/min) **(J)** co-variance of heart rate. For panels D, E, F, G, H, and I, one-way ANOVA; n=5/group. Data are presented as mean ± SD; **p* < .05, ***p* < .01, ****p* < .001, *****p* < .0001

**Supplementary Figure 9**

(A) Haematological parameters of the animals measured 28 days post sCT administration.
(B) H&E staining of heart, spleen, kidney, lung, liver, and brain from C57BL/6 mice after 28 days of intranasal PBS or sCT treatment at the indicated doses.

**Supplementary Figure 10**

**CTR mutants lose the ability to activate the Hippo pathway, and the impact of the V250 M mutation on ICL2 flexibility**

**(A)** The figure shows the cartoon representation of CTR, and the location of seven mutations denoted by yellow stars. **(B)** The plot shows the percentage inhibition of TEAD-Luc activity for the CTR mutants compared to WT CTR upon sCT treatment (300 nM). **(C)** The plot depicts CRE-luciferase activity in cells transfected with vector control, wild-type CTR, or CTR mutants (A51T, V250M, and A307V) upon stimulation with sCT. **(D)** The figure shows the two planes, i.e., plane-0 (yellow), defined by the c-alpha carbon residues R260, I248, and G241, and plane-1 (cyan), defined by the c-alpha carbon residues R260, I248, and V252. The plots show ICL2 angle distribution in the WT and V250M system with respect to residue **(E)** F253, **(F)** T254, and **(G)** E255.

**Supplementary Figure 11**

**Illustration of R404C and R420C mutant orientation with respect to membrane PIP2 interaction and effect on the conformational shift and interaction with membrane PIP2 due to R420C mutation**

**(A)** The superimposed image shows the orientation of the 8th helix in the WT CTR (orange) and R420C mutant (cyan). **(B)** The plot shows the difference in the angle distribution of the 8th helix with the z-axis in the WT CTR and R420C mutant. **(C)** The plot shows the interaction of the 420th residue with PIP2 lipids of the membrane in the WT CTR and R420C mutant. **(D)** The figure shows the bottom view of residue 404 interacting with PIP2 lipid in WT CTR (R404 in orange and PIP2 lipid in gold) and mutant CTR (C404 in cyan and PIP2 lipid in green). **(E)** The figure shows a side view of the exact system mentioned in Figure D. **(F)** The figure shows the bottom view of the interaction of residue 420 with PIP2 lipid in both WT CTR (R420 in orange and PIP2 in gold) and mutant CTR (C420 in cyan and PIP2 in green). **(G)** The figure shows the side view of the exact system mentioned in Figure F.

**Supplementary Figure 12**

**Illustration and quantification of ECD conformational shift due to the R45Q and P100L mutation**

**(A)** The plot shows the minimum distance between the ECD helix and ECL1 loop for WT CTR and R45Q mutant when bound to CT. **(B)** This plot shows the minimum distance between the ECD helix and ECL2 loop for the previously mentioned systems. **(C)** The plot shows the ECD helix angle with respect to the Z-axis for the systems in the same way as Figure A. **(D)** The plot shows the minimum distance between the ECD helix and ECL1 loop for WT CTR and P100L mutant when bound to CT. **(E)** This plot shows the minimum distance between the ECD helix and ECL2 loop for the previously mentioned systems. **(F)** The plot shows the ECD helix angle with respect to the Z-axis for the systems, the same as Figure D. **(G)** The superimposed image shows the ECD orientation between WT CTR (orange) and R45Q mutant (cyan) when bound to CT (pink). **(H)** The superimposed image shows the ECD orientation between WT CTR (orange) and P100L mutant (cyan) when bound to CT (pink).

**Supplementary Figure 13**

**CTR Expression Levels in different TCGA Datasets.** Violin plots depict normalized CTR mRNA expression across independent TCGA datasets, including RNA-seq, Affymetrix, and Agilent platforms. CTR expression was analyzed with respect to molecular subtypes (classical, mesenchymal, and proneural), G-CIMP status, IDH1 mutation status, and MGMT promoter methylation. Across all datasets, CTR expression was consistently enriched in the mesenchymal subtype, with no significant differences observed based on G-CIMP status, IDH1 mutation, or MGMT promoter methylation.
